# Clitorienolactones and Isoflavonoids of *Clitorea ternatea* Roots Alleviate Stress-Like Symptoms in a Reserpine-Induced Zebrafish Model

**DOI:** 10.3390/molecules26144137

**Published:** 2021-07-07

**Authors:** Muhammad Afiq Ngadni, Muhammad Tayyab Akhtar, Intan Safinar Ismail, Anis Irfan Norazhar, Soo Yee Lee, Maulidiani Maulidiani, Khozirah Shaari

**Affiliations:** 1 Natural Medicines and Products Research Laboratory (NaturMeds), Institute of Bioscience, Universiti Putra Malaysia, Serdang 43400, Malaysia; afiq.ngadni@gmail.com (M.A.N.); tayyabakhtar@hotmail.com (M.T.A.); safinar@upm.edu.my (I.S.I.); anisirfan1512@gmail.com (A.I.N.); daphne.leesooyee@gmail.com (S.Y.L.); maulidiani@umt.edu.my (M.M.); 2 Institute of Industrial Biotechnology, Government College University, Lahore 54000, Pakistan; 3 School of Fundamental Science, University Malaysia Terengganu (UMT), Kuala Nerus 21030, Malaysia

**Keywords:** *Clitorea ternatea*, metabolite profile, clitorienolactones, flavonoids, LCMS/MS, reserpine-induced stress, zebrafish model, novel tank test

## Abstract

*Clitorea ternatea* has been used in Ayurvedic medicine as a brain stimulant to treat mental illnesses and mental functional disorders. In this study, the metabolite profiles of crude *C. ternatea* root extract (CTRE), ethyl acetate (EA), and 50% aqueous methanol (50% MeOH) fractions were investigated using ultrahigh-performance liquid chromatography–diode array detector–tandem mass spectrometry (UHPLC–DAD–MS/MS), while their effect on the stress-like behavior of zebrafish, pharmacologically induced with reserpine, was investigated. A total of 32 compounds were putatively identified, among which, a series of norneolignans, clitorienolactones, and various flavonoids (flavone, flavonol, isoflavone, and isoflavanone) was found to comprise the major constituents, particularly in the EA and 50% MeOH fractions. The clitorienolactones, presently unique to the species, were present in both the free and glycosylated forms in the roots. Both the EA and 50% MeOH fractions displayed moderate effects on the stress-induced zebrafish model, significantly decreasing freezing duration and elevating the total distance travelled and average velocity, 72 h post-treatment. The results of the present study provide further evidence that the basis for the use of *C. ternatea* roots in traditional medicine to alleviate brain-related conditions, such as stress and depression, is attributable to the presence of clitorienolactones and the isoflavonoidal constituents.

## 1. Introduction

Recent statistics released by the World Health Organization (WHO) have revealed that more than 264 people globally have been diagnosed with some form of mental disorder, including depression [1]. Gender-wise, women report higher prevalence rates than men, and children and adolescents aged 15 and below are also affected, albeit at lower rates compared to adults. People suffering from depression experience varying clinical symptoms, such as loss of bodyweight, sleep disturbances, and feelings of indecisiveness, guilt, and irritability, as well as intense fatigue [2,3]. Left untreated, such conditions have also been reported to worsen, even leading to suicidal thoughts. The fact that the causes of mental illness are derived from a multitude of factors, such as physiological, biological, and external environment, further exacerbates the situation, making early diagnosis a challenging process [4]. It is now commonly accepted that chronic stress is closely linked to the development of depressive symptoms, along with pathophysiological alteration in both brain structure and brain function [5,6]. Stress-induced depression has thus become one of the neurological states modelled in animal models, such as rodents, mice, and zebrafish, as a means to better understand the condition [7,8].

Present treatments for mental health problems include the use of antidepressant drugs. However, despite their efficacy, synthetic drugs such as tricyclic antidepressants (TCAs) and monoamine oxidase inhibitors (MAOIs) are associated with numerous side effects, such as sleepiness, weight gain, gastrointestinal issues, and sexual dysfunction [9,10]. Hence, an increasing amount of research has been conducted on plant-based remedies as alternative treatments for alleviating stress and depression. Moreover, because of their decent tolerability, low toxicity, and safety, public interest in these alternative medicines has risen over the past 20 years, both in the United States and globally [11]. 

*Clitorea ternatea*, commonly known as “butterfly pea” or “Asian pigeon wings”, is a plant that belongs to the *Leguminosae* family, under the subfamily *Papilionoideae* [12,13,14]. In Ayurvedic medicine, the leaves and roots of the plant are used to treat body pains, infections, animal stings, fever, and constipation [15]. The plant has also been traditionally used as a tonic for the brain called “Medhya Rasayana”, and functions as a medicine for neurological disorders [16]. The aerial parts of the plant have been reported to exert mild central nervous system (CNS) activity, such as nootropic, anxiolytic, antistress, antidepressant, and anticonvulsant activity [14]. Meanwhile, the roots have been reported to possess pharmacological benefits, such as antidepressant [17], antipyretic [18], anti-inflammatory, and analgesic effects [19]. In addition, *C. ternatea* roots have also been shown to enhance the acetylcholine content of rat brains—in particular, the hippocampus region [20]. This unique property could be responsible for its antiamnesic and memory and learning improvement effects on the rodents [21,22]. Previous studies on the chemical profile of *C. ternatea* roots reported the presence of various classes of compounds, including amino acids—comprising glycine, alanine, valine, leucine, aspartic acid, glutamic acid, arginine, and histidine—triterpenoids—specifically taraxerol and taraxerone—the norneolignans—clitorienolactones A, B, C, and D,—aldehyde—(*Z*)-9,17-octadecadienal, and—a fatty acid—palmitic acid [22,23,24,25]. Furthermore, most of these compounds have exhibited promising psychoactive properties. The norneolignans displayed learning and memory enhancement potential [22], taraxerol displayed significant inhibition on acetylcholinesterase (AChE) activity [26], and the last two compounds, (*Z*)-9,17-octadecadienal and palmitic acid, showed promising monoamine oxidase inhibitory action from molecular docking studies [16]. However, despite these early studies, there remains much to be understood about the chemistry and pharmacology of this medicinal plant, particularly in relation to its therapeutic potential for treating brain-related disorders, such as stress and depression. 

In the last few decades, zebrafish (*Danio rerio*) have emerged as a powerful model for neuroscience research, since their physiology strongly parallels both rodents and humans [27,28]. Moreover, zebrafish possess all of the classical neurotransmitters found in vertebrates, suggesting their potential for studying neurodegenerative disorders such as Parkinson’s and Alzheimer’s disease, and mental illnesses such as anxiety and depression. Additionally, zebrafish have a robust and sensitive behavioral response, which could be used as parameters to study neurological effects, such as stress and depression [29]. As part of a continuous search for new pharmacologically active compounds of plant origin, and following the lead provided by its ethnomedicinal use for treating neurological disorders, we embarked on the present study to obtain insights into the biological effects of *C. ternatea* root extract and the chemical constituents responsible for them. The neurological effects of the root extract were tested on zebrafish, which were pharmacologically induced into a stress-like state using reserpine, a drug widely used to mediate depression in experimental animal models. Meanwhile, the chemical constituents of the root extract and its bioactive fractions were profiled via UHPLC–DAD–MS/MS analysis in order to gain insights into the potential active constituents. 

## 2. Results and Discussion

### 2.1. UHPLC–DAD–MS/MS Profiling of C. ternatea Crude Root Extract in Negative Ionization Mode

**Figure 1:**
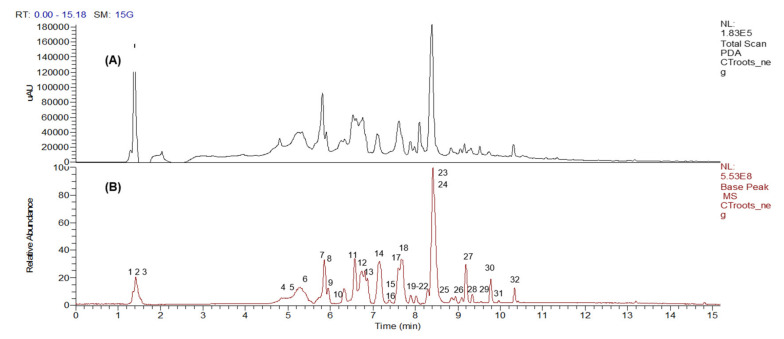
The LC–DAD (**A**) and LC–MS (**B**) base peak chromatogram profiles of *Clitorea ternatea* crude root extract in negative ion mode.

Peak assignments and the metabolites putatively identified in *C. ternatea* root extract based on the LC–MS/MS data obtained in the negative mode (ESI^−^) are displayed in Figure 1 and Table 1, respectively. The base peak chromatogram (Figure 1) showed that most of the compounds were eluted between the 5th and 11th minutes. A total of 32 compounds were identified, as listed according to their different classes in Table 1. The metabolites were characterized based on comparing their fragmentation patterns to those reported in literature and natural products databases. 

#### 2.1.1. Clitorienolactones

**Figure 2:**
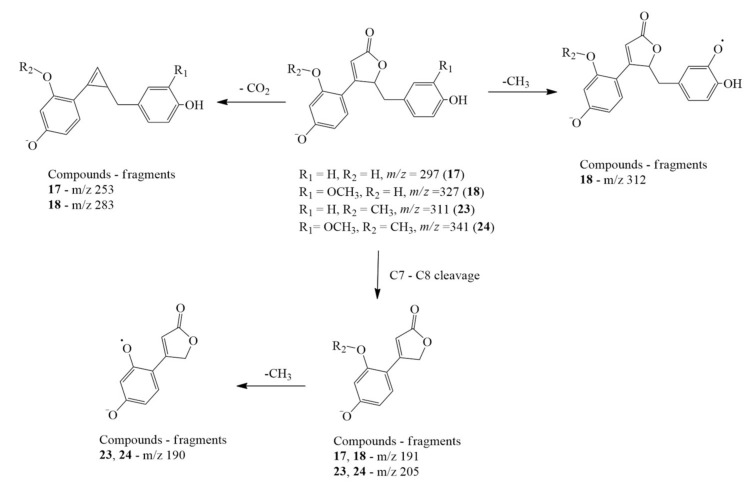
Proposed characteristic fragmentation pathway of compounds **17**, **18**, **23,** and **24** (Clitorienolactones D, C, B, and A, respectively).

The clitorienolactones were detected in the root extracts in both free and glycosylated forms. The free aglycones were detected at *m/z* 297.0754, 327.0858, 311.091, and 341.1013, which were the deprotonated molecular ions for peaks **17**, **18**, **23,** and **24**, respectively. The compounds were characterized as clitorienolactones D, C, B, and A, based on comparison with reported MS data [22]. The fragmentation patterns of these compounds are illustrated in detail in Figure 2, showing characteristic losses of CO_2_ (−44 amu) from the lactone ring and carbon cleavage at C7–C8. Such a fragmentation pathway is not uncommon, as it has been reported in mass fragmentation dibenzylbutyrolactone lignans [39]. In addition, losses of CH_3_ (−15 amu) were also detected where the methyl group was cleaved via radical fragmentation. Moreover, the UV spectra of these clitorienolactones matched those reported in previous reports, as shown in Supplementary Materials Figure S1.

Peaks **11**, **12**, **13**, and **14**, with deprotonated molecular ions at *m/z* 459.1278, 489.1383, 503.1537, and 473.1429, respectively, were assigned as the O-hexosides of the clitorienolactones, identified as clitorienolactone D-O-hexoside, clitorienolactone C-O-hexoside, clitorienolactone A-O-hexoside, and clitorienolactone B-O-hexoside, respectively. The presence of Y_0_^−^ ([M-H-C_6_H_9_O_5_]^−^) ions in their MS/MS spectra corresponded to the loss of a single hexose unit. Meanwhile, peaks **5**, **6**, **7,** and **8**, with deprotonated molecular ions at *m/z* 621.1796, 651.1931, 635.1950, and 665.2057, respectively, exhibited typical fragmentation of O-dihexosides via losses of 162 amu. They were identified as clitorienolactone D-O-dihexoside, clitorienolactone C-O-dihexoside, clitorienolactone B-O-dihexoside, and clitorienolactone A-O-dihexoside, respectively. The formation of Y_1_^−^ ([M-H-C_6_H_9_O_5_]^−^) and Y_0_^−^ ([M-H-C_6_H_9_O_5_-C_6_H_9_O_5_]^−^) ions in their MS/MS fragmentation (Table 1) was due to the loss of the terminal sugar (hexoside) units and rearrangement reactions of the interglycosidic bonds [40,41].

#### 2.1.2. Flavonoids

Several classes of flavonoids were identified in the root extract, comprising isoflavones (peaks **19**, **21**, **25,** and **29**), a flavonol (**20**), flavones (**22, 26**, and **27**), an isoflavanol (**28**), and isoflavanons (**30**, **31,** and **32**). Identification of the four isoflavones was based on their characteristic fragmentation patterns resulting from retro-Diels–Alder (RDA) reactions and successive losses of CO molecules. The identified isoflavones were: daidzein (peak **19**), with a deprotonated molecular ion at *m/z* 253.0493, and fragment ions at *m/z* 224 ([M-H-CO-H]^•−^), 196 ([M-H-CHO-CO]^•−^), 135 (^1,3^A^−^), 133 (^0,3^B^−^), and 117 (^1,3^B^−^); glycitein (peak **21**), with a deprotonated molecular ion at *m/z* 283.0598, and fragment ions at *m/z* 268 ([M-H-CH_3_]^•−^), 240 [M-H-CH_3_-CO]^•−^), 239 [M-H-CH_3_-CO-H]^−^), 212 [M-H-CH_3_-CO-CO]^•−^)s and 211 [M-H-CH_3_-CO-H-CO]^−^); genistein (peak **25**), with a deprotonated molecular ion at *m/z* 269.0444, and fragment ions at *m/z* 241 ([M-H-CO]^−^), 213 ([M-H-CO-CO]^−^), 135 (^0,3^A^−^)s and 133 (^0,3^B^−^); and, lastly, formononetin (peak **29**), with a deprotonated molecular ion at *m/z* 267.0651), and fragment ions at *m/z* 252 ([M-H-CH_3_]^•−^), 224 ([M-H-CH_3_-CO]^•−^), 195 ([M-H-CH_3_-CO-H-CO]^−^), 135 (^1,3^A^−^), and 132 (^0,3^B^−^). Not only are these fragmentation patterns and their peak assignments in accordance with previous findings [33,38], the order of elution of these isoflavones also tallies with those of published reports [42,43]. Comparison with the standards also confirmed the identification of peaks **19**, **25,** and **29** as daidzein, genistein, and formononetin, respectively, as shown in Supplementary Materials Figure S2. Peak **21** was initially assumed to be biochanin A; however, the dissimilarity in retention time disapproved this assumption, thus strengthening its identification as glycitein, an isomer of biochanin A. 

Peak **20**, with a deprotonated molecular ion at *m/z* 297.0753, was identified as 3′,4′-dimethoxyflavonol, based on the accompanying fragment ions at *m/z* 282 ([M-H-CH_3_]^−^), 267 ([M-H-CH_3_-CH_3_]^−^), 254 ([M-H-CH_3_-CO]^−^), and 239 ([M-H-CH_3_-CO-CH_3_]^−^), all of which were consistent with data from the online database [30]. One of the three flavones detected was identified as diosmetin (peak **22**), with a deprotonated molecular ion at *m/z* 299.0546, and fragment ions at *m/z* 284 ([M-H-CH_3_]^−^), 271 ([M-H-CO]^−^), and 256 ([M-H-CO-CH_3_]^−^). The luteolin derivative, luteolin-3′,4′-dimethyl ether, was assigned to peak **26**, and displayed a deprotonated molecular ion at *m/z* 313.0704, and fragment ions at *m/z* 298 ([M-H-CH_3_]^−^), 283 ([M-H-CH_3_-CH_3_]^−^), 270 ([M-H-CH_3_-CO]^•−^), 255 ([M-H-CH_3_-CO-CH_3_]^−^), and 239 ([M-H-CH_3_-CO-OCH_3_]^−^). The third flavone, hoslundal, was assigned to peak **27**, with a deprotonated molecular ion at *m/z* 309.0752), and fragment ions at *m/z* 294 ([M-H-CH_3_]^−^), 266 ([M-H-C_2_H_3_O]^−^), 222 ([M-H-C_2_H_3_O-CO_2_]^−^), 249 ([M-H-CH_3_-CO_2_H]^−^), 148 ([0,3A-CH_2_CO]^−^), and 132 ([^0,4^A-CH_2_O]^−^). These identifications consistent with data from the online database and previous literature reports [30,44]. The proposed fragmentation pathways of compounds **20**, **22**, **26,** and **27** are illustrated in Supplementary Materials Figures S3–S6.

Peaks **30** and **32**, which exhibited similar molecular ions at *m/z* 353.1013 and similar fragmentation patterns, were identified as ambonone and its isomer. Their fragment ions appeared at *m/z* 338 ([M-H-CH_3_]^−^), 323 ([M-H-CH_3_-CH_3_]^−^), 308 ([M-H-CH_3_-CH_3_-CH_3_]^•−^), 293 ([M-H-CH_3_-CH_3_-CHO]^−^), and 279 ([M-H-CH_3_-CH_3_-CH_3_-CHO]^−^). Lastly, peak **31** was identified as neoraunone, an isoflavanone with a similar structure to ambonone, but with only 2 methoxy groups attached to the B-ring; it displayed a deprotonated molecular ion at *m/z* 323.0911, and fragment ions at *m/z* 308 ([M-H-CH_3_]^−^), 293 ([M-H-CH_3_-CH_3_]^−^), 265 ([M-H-CH_3_-CH_3_-CO]^−^), 249 ([M-H-CH_3_-CH_3_-CO_2_]^−^), 237 ([M-H-CH_3_-CH_3_-CO-CO]^−^), and 221 ([M-H-CH_3_-CH_3_-CO_2_-CO]^−^). The proposed fragmentation pathways for ambonone isomers and neoraunone are illustrated in Supplementary Materials Figures S7 and S8, respectively. 

Three isoflavonoid glycosides were identified: genistin, daidzin, and glycitin (peaks **9**, **10,** and **16**, respectively), with molecular ions at *m/z* 431.0963, 415.1016, and 445.1124, respectively. The MS/MS spectra showed that the formation of [Y_0_–H]^−•^ ions in compounds **9** and **10** (*m/z* 268 and 252) was at relatively higher abundance compared to their respective Y_0_^−^ ions (*m/z* 269 and 253) (Table 1). This fragmentation behavior, and the formation of these ions, which were formed through a loss of glucose + H (−163 amu) and glucose (−162 amu) moiety respectively, are in agreement with those of genistin and daidzin [41]. Meanwhile, the MS/MS spectra of compound **16** displayed a base peak at *m/z* 283 (Y_0_^−^ ion) for loss of glucose (−162 amu) from the parent ion, and characteristic fragment ions of the aglycone unit, such as *m/z* 240 [M-H-CH_3_-CO]^•−^), 239 [M-H-CH_3_-CO-H]^−^), and 133 (^0,3^B^−^), all of which suggested it to be glycitin [37,38].

#### 2.1.3. Identification of Amino Acids and Carboxylic Acids

Peaks **1**, **2**, **3,** and **4**, showing molecular ions at *m/z* 154.0605, 173.1027, 131.0445, and 203.0811, respectively, were identified as amino acids. Peak **1,** with fragment ions at *m/z* 137 ([M-H-NH_2_]^−^) and 110 ([M-H-CO_2_]^−^), was identified as histidine, while peak **2**, with a single fragment ion at *m/z* 131 ([M-H-CH_3_N_2_]^−^), was identified as arginine. Peak **3,** with fragment ions appearing at *m/z* 114 ([M-H-NH_3_]^−^), 113 ([M-H-H_2_O]^−^), 72 ([M-2H-C_2_H_3_NO]^−^, 70 ([M-2H-CHNO_2_]^−^), and 58 (M-H-C_2_H¬_2_NO_2_]^−^), was attributed to asparagine. Peak **4** was identified as tryptophan (*m/z* 203.0811), displaying characteristic fragment ions at *m/z* 159 ([M-H-CO_2_]^−^), 142 ([M-H-CH_3_NO_2_]^−^), and 116 [22,23,24]. Meanwhile, azelaic acid, a dicarboxylic acid, was assigned as peak **15** (*m/z* 187.0959), displaying fragment ions at *m/z* 125 ([M-H-CH_2_-O_3_]^−^), *m/z* 97 ([M-H-C_3_H_6_O_3_]^−^), and *m/z* 57 ([M-H-C_6_H_10_O_3_]^−^) [30,32].

### 2.2. UHPLC–DAD–MS/MS (Positive Ionization Mode) Profile of Clitorea ternatea Crude Root Extract 

Several amino acids were exclusively identified in the positive ionization mode where their peaks appeared in the first 5 min. These amino acids were identified as: proline (*m/z* 116.0695), with a single fragment ion at *m/z* 70 ([M+H-H_2_O-CO]^+^); isoleucine (*m/z* 132.1005), with fragment ions appearing at *m/z* 86 ([M+H-H_2_O-CO]^+^) and 69 ([M+H-H_2_O-CO-NH_3_]^+^); and phenylalanine (*m/z* 166.0844), with key fragment ions at *m/z* 149 ([M-H-NH_3_]^+^), 120 ([M+H-H_2_O-CO]^+^), 131 ([M+H-NH_3_-H_2_O]^+^), and 103 ([M+H-NH_3_-H_2_O-CO]^+^). The identification of these amino acids was consistent with previous published reports [30,31,32,45,46,47].

Furthermore, positive ionization allowed the identification of the isoflavanol ambanol (peak **28**), which exhibited a protonated molecular ion at *m/z* 341.0979, and fragment ions at *m/z* 323 ([M-H-H_2_O]^−^), 308 ([M+H-H_2_O-CH_3_]^+^), 295 ([M+H-H_2_O-CO]^+^), 281 ([M+H-H_2_O-CH_2_O]^+^), and 263 ([M+H-H_2_O-CO-CH_3_OH]^+^). Based on these spectral data, the fragmentation pathway was deduced using Mass Frontier 3.0 software, and is illustrated in Supplementary Materials Figure S9. Additionally, the clitorienolactones (**17**, **18**, **23,** and **24**), clitorienolactone glycosides (**7**, **11**–**14**), flavonoids (**19**–**21**, **26**–**31**), and flavonoid glycoside (**16**) were also detected in positive ion mode. The MS/MS data that listed their fragment ions and their peak assignments are shown in Supplementary Materials Table S1 and Figure S10.

### 2.3. Metabolite Composition of the Ethyl Acetate and 50% MeOH Fractions

**Figure 3:**
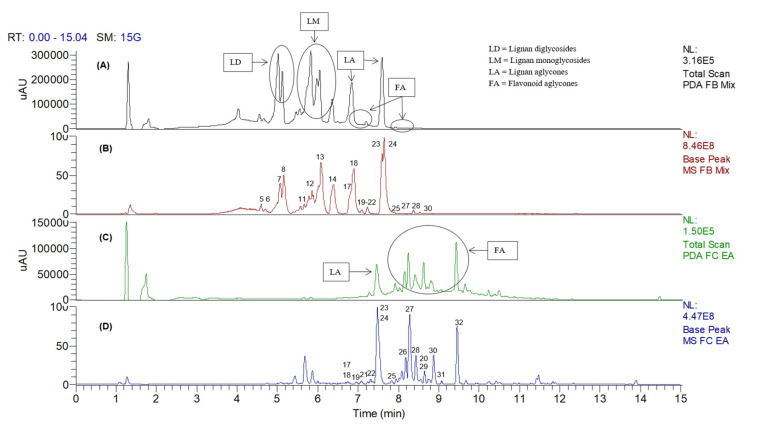
The LC–DAD and LC–MS base peak chromatogram profiles of the 50% MeOH (**A**,**B**) and EA (**C**,**D**) fractions of *Clitorea ternatea* roots, in negative ion mode.

To further investigate the distribution of metabolites across fractions of varying solvent polarities, the crude extract of *C. ternatea* root was fractionated into several fractions using SPE. In contrast to the ethyl acetate and 50% methanol fractions, the yields of the aqueous and hexane fractions were too small for subsequent bioactivity, as depicted in Supplementary Materials Table S2. Therefore, only the ethyl acetate and 50% MeOH fractions were subjected to UHPLC–DAD–MS/MS analysis. The chemical profiles of both fractions in negative ion mode, and the relative abundance of the metabolites, are illustrated in Figure 3. It could be observed that, although the major free lignans, clitorienolactones A (**24**) and B (**23**), were present in both fractions, the norneolignans and their respective glycosides, (**5**–**8**, **11**–**14**) were more soluble in the 50% MeOH fraction (Figure 3A,B). On the other hand, the flavonoidal constituents were more soluble in the EA fraction (Figure 3C,D). 

### 2.4. Effect on Reserpine-Induced Stress in Zebrafish 

**Figure 4:**
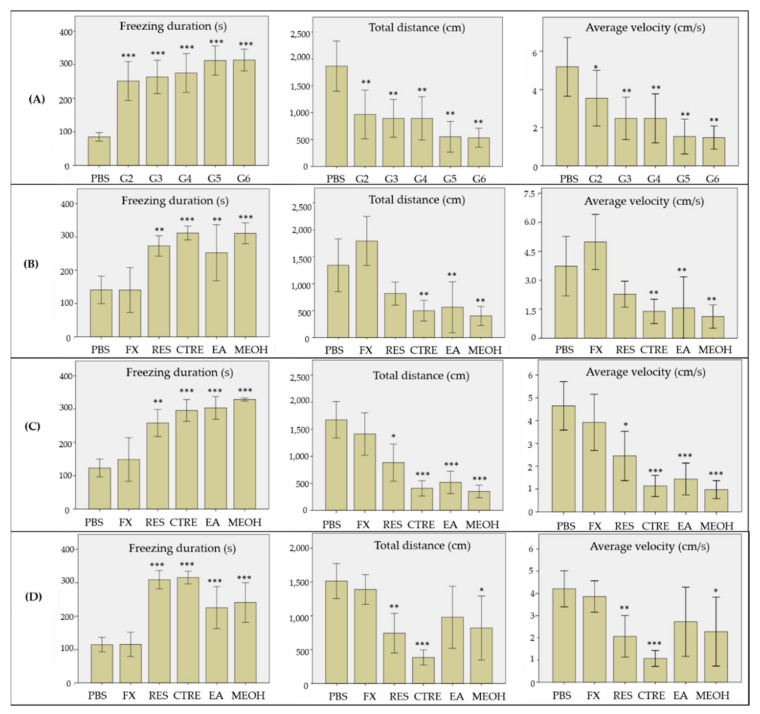
Behavioral effects of the crude methanolic extract, EA, and 50% MeOH fractions of *Clitorea ternatea* roots on the reserpine-induced stress zebrafish model. Behavioral measurements were recorded at (**A**) 24 h after IP injection of reserpine, (**B**) 24 h after treatments, (**C**) 48 h after treatments, and (**D**) 72 h after treatments for each group, i.e., fluoxetine (0.6 mg/mL, 30 min), CTRE, EA, and 50% MeOH fractions groups (35 mg/mL, 6 h). All values are reported as mean percentage ± SE (*n* = 10 per group). Duncan’s post-hoc test was used for significant differences, where * = *p* < 0.05, ** = *p* < 0.01, and *** = *p* < 0.001. PBS: phosphate buffer saline; FX: fluoxetine; RES: reserpine; CTRE: crude *C. ternatea* extract; EA: ethyl acetate; MEOH: 50% methanol.

The effect of the crude MeOH and solvent fractions of *C. ternatea* roots was evaluated on a reserpine-induced stress zebrafish model, developed and optimized according to our previous work [48]. The behavioral changes of the model were observed using the novel tank test. Several parameters were monitored and recorded as measurements for the stress-like behavior, consisting of freezing duration, total distance travelled, and average velocity. The effects of the crude extract and solvent fractions on the selected behavior parameters are presented in Figure 4. As shown in Figure 4A, the model groups 2–6 exhibited significantly longer freezing duration, lower total distance travelled, and reduced average swimming velocity, compared to the control group (PBS). These results indicate that reserpine treatment has successfully induced stress-like behavior in the zebrafish, consistent with previous findings, where reserpine treatment produced diminished motor coordination and reduced exploratory behavior in zebrafish. Diminished motor coordination and reduced exploratory behavior are also among the accepted symptoms for depression [49]. Treatment of the model with fluoxetine (positive control) showed reduction in freezing duration, and increments in the total distance travelled and average velocity, with values comparable to those of the control group (*p* > 0.05), 24 h after the treatment (Figure 4B). Fluoxetine has been commonly used in similar research as a positive standard drug, to alleviate novelty stress and anxiety in zebrafish, and to test the predictive validity of the animal model [50,51]. The behavioral changes shown by the fluoxetine-treated group showed that the stress-like behavior in the zebrafish was reversible after exposure to the drug.

In contrast to the positive control, the stress-like behavior in groups treated with the crude methanolic extract and solvent fractions of *C. ternatea* roots persisted up to 48 h post-treatment (Figure 4C). In fact, the group treated with the crude methanolic extract showed no signs of recovery, and remained in the stress-like state even up to 72 h post-treatment. However, at 72 h post-treatment, there were clear behavioral changes observed in groups treated with the EA and 50% MeOH fractions, as shown in Figure 4D. The freezing duration of both groups decreased significantly in comparison to the untreated group (*p* < 0.05), although the durations remained statistically higher those that of the control and fluoxetine-treated groups (*p* < 0.001). The total distance travelled and average swimming velocity values of both groups were also significantly increased, with values higher than the untreated group (*p* < 0.05). It is also worthy of note that the group treated with the EA fraction displayed more pronounced recovery in mobility and exploratory behavior, where their swimming velocity and the distance travelled were comparable to those of the normal and fluoxetine-treated groups (*p* > 0.05). In contrast, the behavioral parameters were improved to a lesser extent for the groups treated with the 50% MeOH fraction, where the distance travelled and average swimming velocity values were still significantly lower than those of the control group (*p* < 0.05). The results suggest that the stress-relieving constituents of *C. ternatea* roots are mainly those present in the EA and 50% MeOH fractions. Based on the metabolite profile of the EA fraction obtained via UHPLC–DAD–MS/MS analysis, the bioactivity could be attributed to the presence of both the free clitorienolactones and the flavonoids, since these compounds were detected in higher abundance compared to other compounds in the EA fraction. On the other hand, bioactivity of the 50% MeOH fraction could be attributable to the presence of the free clitorienolactones and their O-glycosides, since they were relatively more abundant in this fraction compared to the flavonoids.

Clitorienolactones A and B have previously been found to be responsible for learning and memory enhancement via the inhibition of acetylcholinesterase (AChE), the enzyme responsible for disrupting the transmission of nerve impulses in the cholinergic system [22,52]. Similarly, to vertebrates, acetylcholine in the cholinergic system of zebrafish plays a key role in both CNS functions (sleeping, attention, learning, and memory) and CNS dysfunctions (immobility, lack of coordination, depression, and memory loss) [53]. For instance, physostigmine, an AChE inhibitor, demonstrated anxiolytic effects [54] and caused the recovery of learning deficits [55] in zebrafish. Henceforth, the ability of clitorienolactones to elevate acetylcholine content in zebrafish brains by suppressing AChE activity may also work in favor of relieving stress-like symptoms. However, several studies have found contradicting results on the effects of AChE inhibition on murine models of depression. Behavioral studies have discovered that enhancing ACh neurotransmission in the hippocampus via AChE inhibition is enough to evoke anxiety- and depression-like symptoms in rodents, which were later alleviated through fluoxetine administration [56,57,58]. Similar findings have also been reported for adolescents who have been exposed to substantial amounts of cholinesterase inhibitors in agricultural pesticides [59]. The cholinergic–adrenergic theory of depression may explain these results, where overactivity of the cholinergic system over the adrenergic system was hypothesized to lead to depression [60]. Therefore, some researchers concluded that maintaining a homeostatic level of ACh in the hippocampus is crucial to controlling emotional behavior [57]. Due to their ability in inhibiting AChE, we proposed that the clitorienolactones A and B played a part in maintaining a homeostatic ACh level in the brains of the zebrafish, particularly in preventing ACh depletion. However, further research is still required in order to understand the neuropharmacological effects of these lignans and their derivatives on both the monoamine and cholinergic systems in the suitable models of stress and depression.

In addition to the norneolignans, several isoflavonoids (daidzein, genistein, glycitein, and formononetin) were also present in relatively small amounts in both the EA and 50% MeOH fractions. Daidzein and genistein are known constituents of pomegranate extract, which have been shown to exert antidepressant effects on menopause-related depression in ovariectomized mice [61]. Meanwhile, in a separate study, genistein was shown to have the ability to evoke antidepressant-like effects in rats by adjusting their hippocampal serotonergic metabolism when stressed [62]. Formononetin has also been identified as one of the active compounds with an effective inhibitory effect against monoamine oxidase, an enzyme responsible for the degradation of monoamine neurotransmitters such as serotonin, dopamine, and norepinephrine [63]. Thus, we hypothesize that the ability of these isoflavonoids to modulate serotonergic function may explain the plant’s efficacy in alleviating stress-like behavior in the zebrafish model. Further studies into the effect of this class of compounds are therefore needed in order to confirm this hypothesis, in addition to further investigation into the possible synergistic effects between the clitorienolactones, isoflavones, and other minor constituents detected in the bioactive fractions.

## 3. Materials and Methods

### 3.1. Chemicals and Materials

LCMS-grade methanol, acetonitrile, water, and formic acid were purchased from Fisher Scientific (Hampton, NH, USA), while reserpine (>99%, HPLC-grade) and fluoxetine hydrochloride were purchased from Sigma-Aldrich (St. Louis, MO, USA). The standards, daidzein (≥98%), genistein (≥98%), biochanin A (≥98%), and formononetin (≥98%) were purchased from ChemFaces (Wuhan, China). Phosphate buffer saline (PBS) buffer (pH 7.4) was purchased from R&M Chemicals (Petaling Jaya, Malaysia). The solid-phase extraction (SPE) cartridge used was a silica-based Chromabond C18 polypropylene column (MACHEREY-NAGEL GmbH & Co. KG, Düren, Germany). Holding water for the zebrafish during acclimatization and for experimental procedures was sourced from Coway-filtered tap water treated with water conditioner (Tension gon, Mydilab, Pulau Pinang, Malaysia). The syringe and needle (Ultra-Fine II, 0.30 mm (30 G) × 8 mm (5/16″), 0.5 mL) used for intraperitoneal (IP) injection were obtained from Becton Dickinson, Franklin Lakes, NJ, USA. All tanks used were standard plastic tanks (3 L, 5 L, and 7 L) bought from 3B Aquatics, Bangi, Malaysia.

### 3.2. Plant Material

Whole plants of *Clitorea ternatea* were purchased from a plant nursery in Sungai Buloh, Selangor, Malaysia. Species authentication was performed by the Biodiversity Unit at the Institute of Bioscience, and a voucher specimen (SK 3297/18) was deposited at the Unit’s mini-herbarium. The roots were harvested from each plant, cleaned, and dried under shade for 7 days. The dried roots were cut into small pieces, ground into a fine powder, and stored in a vacuum desiccator until further use.

### 3.3. Solvent Extraction of Dried Roots

For extraction, the dry powder was weighed (56 g), mixed with methanol at a solid:liquid ratio of 1:20 (g:mL), and sonicated (53 kHz) for 30 min, with the temperature maintained between 27 and 30 °C. After solvent removal, the extract was further concentrated in a rotary evaporator and subjected to freeze-drying over three days in order to completely remove any moisture. The resulting process yielded a light brown, powdered extract (7.84 g, 14% yield), labelled “CTRE”.

### 3.4. Fractionation of Crude Extract

The methanolic extract of *C. ternatea* roots was subjected to solid-phase extraction (SPE) using C18 SPE cartridges (60 Å pore size, 45 um particle size, 6 mL volume, 1 g adsorbent weight) to give four fractions, namely, aqueous, 50% methanol, ethyl acetate, and hexane fractions. The cartridge was first activated using methanol (LCMS grade, 3 mL). Next, deionized water (6 mL) was used to remove excess MeOH by applying a gentle vacuum. The extract (20 mg) was reconstituted in 1 mL water prior to the SPE. After sample loading, the cartridge was eluted with deionized water, followed by 50% aqueous MeOH, ethyl acetate, and then hexane. The eluents were collected in test tubes, and after solvent removal, the weight and yield of each solvent fraction were recorded. 

### 3.5. UHPLC–DAD–MS/MS Analysis

The UHPLC–DAD–MS/MS analysis was conducted using a Dionex Ultimate 3000 UHPLC system coupled with a Thermo Scientific™ Q Exactive™ Hybrid Quadrupole-Orbitrap mass spectrometer (Thermo Fisher Scientific, Bremen, Germany). The UHPLC system was equipped with a diode array detector (DAD), scanning from 190 to 800 nm. Crude extract and fraction samples for analysis were prepared in methanol at 2 mg/mL and filtered through a 0.22 µm nylon membrane. Meanwhile, another crude extract sample (1 mL) was spiked with 100 μL of standards prepared at 0.135 mg/mL for qualitative analysis. Analyte separation was performed using a reversed-phase Acquity UPLC^®^ BEH C18 column (100 mm × 2.1 mm × 1.7 um) (Waters, Ireland) with a gradient mobile phase consisting of LCMS-grade acetonitrile (solvent A) and water (solvent B), each containing 0.1% formic acid. The gradient system consisted of 5% solvent A in the first 0.3 min, followed by 5–80% A in the next 15 min. The flow rate of the mobile phase was set at 0.5 mL/min, while the injection volume was 10 µL. Meanwhile, the UV detector was set to 260, 254, 310, and 350 nm while the PDA detector was set to 190–800 nm. The MS/MS parameters were set as follows: negative and positive ion mode (done separately), collision energy of 30 eV, spray voltage 4.2 kV, capillary temperature of 320 °C, auxiliary gas heater temperature of 0 °C, sheath gas flow rate of 40 arbitrary units, and auxiliary nitrogen (99% pure) gas unit flow rate of 8. The mass resolution was set to 70,000 full widths at half maximum (FWHM), and the scan ranged between 102 and 1532 amu. All chromatographic methods were conducted at room temperature, and the resulting peaks from the tandem mass spectrometry were analyzed and annotated using Thermo Xcalibur 2.0 software (Thermo Fisher Scientific Inc., Waltham, MA, USA) and compared with information from previous literature and online databases.

### 3.6. Zebrafish and Maintenance

Adult zebrafish (4–5 months old) of common wild-type (WT) stock (short-fin phenotype) were purchased from a local supplier (3B Aquatics, Bangi, Selangor, Malaysia). The fish were bred under standard husbandry conditions, maintained in treated filtered water at room temperature 28 °C, pH 7.0, and in a 14 h light and 10 h dark cycle (light onset: 8 a.m.; offset: 10 p.m.). The fish were fed with a commercial flake fish food (Ziegler, Gardners, PA, USA) three times per day, ad libitum. The standard fish tanks were equipped with air pumps in order to ensure constant aeration and to maximize oxygen content in the holding water. The fish were acclimatized under lab conditions at least 7 days prior to experiments.

### 3.7. Induction of Stress

**Figure 5:**
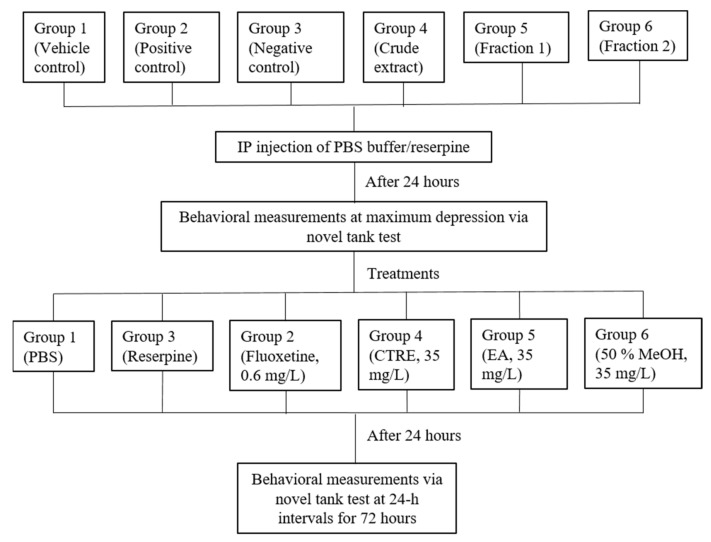
Groupings and experimental workflow of the zebrafish in vivo assay.

Induction of stress in zebrafish was carried out based on previously published research [48,64]. The fish were divided into 6 groups of 10 fish (5 males; 5 females). Group 1 was given PBS buffer at 5 uL/g bodyweight (BW), serving as the vehicle control. Intraperitoneal (IP) injections of reserpine (20 mg/kg BW) were administered to Groups 2–6 in order to induce stress-like responses. Before carrying out the induction, the acclimatized fish underwent fasting for 24 h. Prior to injection, the fish were anesthetized by immersion in a bath of iced water (12 °C), until operculum movement ceased or until only the gills were moving [65]. Treatments were then administered to the stress-induced fish via the immersion method, 24 h post-injection. Group 2 (positive control) was treated with 30 min immersion in 0.6 mg/L fluoxetine solution. Group 3 (negative control) received no treatment. Test sample treatment groups 4, 5, and 6 were treated with 6 h of immersion in 35 mg/L of the CTRE (Group 4), EA (Group 5), and 50% MeOH (Group 6) fractions, respectively. Fluoxetine and test sample solutions were prepared using Milli-Q water prior to immersion treatment. The fish groupings and workflow for the in vivo assay are illustrated in Figure 5. The experimental setup and procedures were approved by the Institutional Animal Care Use Committee (IACUC) of Universiti Putra Malaysia on 10 June 2015 (AUP-R013/2015).

### 3.8. Behavioral Assay: Novel Tank Test

Twenty-four hours after reserpine-induced stress, the animals were individually submitted to the novel tank test for behavioral measurements, carried out according to a previously developed method [50]. For the measurement, the fish were carefully placed in individual 3 L tanks (novel tanks) filled with holding water (2 L) for 6 min. The novel tanks were rested on a flat platform, bottom-lit with white light, and white cardboard was placed between the tanks in order to prevent visual contact with fish from neighboring tanks. The water in the tanks was changed for each test. Once placed in the novel tank, the swimming pattern and movement of the fish were video recorded and tracked using ZebraLab software (Viewpoint, Civrieux, Rhone-Alpes, France). The following endpoints were measured: freezing duration (s), average velocity (cm/s), and total distance travelled (cm). Freezing is defined as complete absence of fish movement except for the eyes and gills for a period of 1s or longer [50,66]. On the other hand, the total distance travelled and average velocity are measurements based on the total distance and speed with which the fish swam over the 6 min test duration. Subsequent behavioral measurements were taken at every 24-h interval, up to 72 h post-treatment.

### 3.9. Statistical Analysis

Data acquired from the novel tank test were analyzed using SPSS Statistics 17.0 software. Analysis of variance (ANOVA) was performed on the experimental data, and the means were compared using Duncan’s multiple range test.

## 4. Conclusions

The metabolome of *C. ternatea* roots was putatively profiled using UHPLC–PDA detection and ESI–MS/MS analysis. A total of 32 metabolites, comprising lignans, lignan glycosides, flavonoids, flavonoid glycosides, amino acids, and carboxylic acids, were identified in the crude methanol extract. Chemical profiling of the fractions revealed that both the EA and 50% methanol fractions contained the free clitorienolactones. However, the EA fraction was found to be richer in the flavonoidal constituents, while the 50% MeOH fraction was richer in the clitorienolactone glycosides. Both the EA and 50% MeOH fractions exhibited moderate effects on stress-induced zebrafish, which were concluded to be due to the combined effects of the clitorienolactones and flavonoidal constituents, specifically the isoflavonoids. The results of the present study highlight the potential therapeutic value of *C. ternatea* roots in treating brain-related conditions, such as stress, anxiety, and depression, which thus merit further research for a deeper understanding of their mode and mechanism of action. Such studies should be performed on the isolated bioactive constituents, or in the form of standardized botanical extract preparation, with the aim of realizing *C. ternatea’s* potential as a drug lead, or its development as a phytomedicine for mental healthcare. 

## Figures and Tables

**Table 1:** Metabolites putatively identified from the LC–MS/MS (negative ion mode) spectral data of *Clitorea ternatea* root extract.

No	Retention Time (RT) (min)	Parent Ion Experimental (*m/z*)	Parent Ion Theoretical (*m/z*)	Error (ppm)	MS/MS Fragment Ions (Intensity, %)	Tentative Identification	Molecular Formula	Source
Amino Acids and Carboxylic Acids								
1	1.24	154.0605	154.0615	–6.49	154 (100), 137 (42), 110 (15)	Histidine	C_6_H_9_N_3_O_2_	[30]
2	1.31	173.1027	173.1038	−6.35	131 (100)	Arginine	C_6_H_14_N_4_O_2_	[30]
3	1.36	131.0445	131.0456	−8.39	131 (19), 114 (100), 113 (62), 95 (14), 88 (32), 72 (15), 70 (40), 58 (10)	Asparagine	C_4_H_8_N_2_O_3_	[31]
4	4.82	203.0811	203.0820	−4.43	203 (72), 159 (20), 142 (28), 116 (100)	Tryptophan	C_11_H_12_N_2_O_2_	[30,32]
15	7.38	187.0959	187.0970	−5.87	187 (47), 125 (100), 97 (8), 57 (3)	Azelaic acid	C_9_H_16_O_4_	[30,32]
Clitorienolactones								
17	7.59	297.0754	297.0763	−3.03	297 (20), 253 (100), 191 (5) 159 (10), 133 (20), 119 (34), 109 (23), 93 (20)	Clitorienolactone D	C_17_H_14_O_5_	[22]
18	7.66	327.0858	327.0868	−3.06	327 (26), 283 (67), 267 (100), 161 (11), 159 (14), 109 (6)	Clitorienolactone C	C_18_H_16_O_6_	[22]
23	8.41	311.0910	311.0919	−2.89	311 (32), 205 (74), 191 (10), 190 (100), 161 (9)	Clitorienolactone B	C_18_H_16_O_5_	[22]
24	8.47	341.1013	341.1025	−3.51	341 (42), 205 (68), 191 (7), 190 (100), 161 (7)	Clitorienolactone A	C_19_H_18_O_6_	[22]
Clitorienolactone Glycosides								
5	5.29	621.1796	621.1819	−3.70	621 (6), Y_1_^−^: 459 (25), Y_0_^−^: 297 (19), 253 (100)	Clitorienolactone D 4-O-dihexoside	C_29_H_34_O_15_	-
6	5.84	651.1931	651.1925	0.92	651 (5), Y_1_^−^: 489 (35), Y_0_^−^: 327 (35), 283 (100), 268 (54)	Clitorienolactone C 4-O-dihexoside	C_30_H_36_O_16_	-
7	5.85	635.1950	635.1976	−4.09	Y_1_^−^: 473 (31), Y_0_^−^: 311 (100), 205 (79), 190 (20)	Clitorienolactone B 4-O-dihexoside	C_30_H_36_O_15_	-
8	5.95	665.2057	665.2081	−3.61	Y_1_^−^: 503 (33), Y_0_^−^: 341 (100), 205 (65),190 (16)	Clitorienolactone A 4-O-dihexoside	C_31_H_38_O_16_	-
11	6.53	459.1278	459.1291	−2.83	459 (8), Y_0_^−^: 297 (43), 253 (100),109 (8)	Clitorienolactone D 4-O-hexoside	C_23_H_24_O_10_	-
12	6.63	489.1383	489.1396	−2.66	489 (6), Y_0_^−^: 327 (83), 283 (100), 268 (67)	Clitorienolactone C 4-O-hexoside	C_24_H_26_O_11_	-
13	6.85	503.1537	503.1553	−3.18	Y_0_^−^: 341 (100), 205 (80), 190 (52), 161 (4)	Clitorienolactone A 4-O-hexoside	C_25_H_28_O_11_	-
14	7.15	473.1429	473.1447	−3.8	473 (18), Y_0_^−^: 311 (96), 205 (100), 190 (49), 161 (5)	Clitorienolactone B 4-O-hexoside	C_24_H_26_O_10_	-
Flavonoids								
19	7.89	253.0493	253.0500	−2.76	253 (2), 224 (10), 223 (38), 208 (32), 196 (8), 195 (41), 180 (23), 167 (8), 135 (8), 133 (36), 132 (81), 117 (8), 91 (100)	Daidzein *	C_15_H_10_O_4_	[33,34]
20	8.01	297.0753	297.0763	−3.36	297 (38), 282 (100), 267 (10), 254 (30), 239 (28), 195 (4)	3′,4′-dimethoxyflavonol	C_17_H_14_O_5_	[30]
21	8.04	283.0598	283.0606	−2.82	268 (100), 267 (2), 240 (6), 239 (27), 212 (1), 211 (7), 184 (3), 148 (2), 135 (1),	Glycitein	C_16_H_12_O_5_	[33,35]
22	8.18	299.0546	299.0555	−3.01	299 (24), 284 (25), 271 (13), 256 (100), 255 (6)	Diosmetin	C_16_H_12_O_6_	-
25	8.76	269.0444	269.0450	−2.23	269 (100), 241 (7), 224 (3), 213 (1), 197 (4), 183 (5), 135 (10), 133 (4)	Genistein *	C_15_H_10_O_5_	[33,36]
26	9.11	313.0704	313.0712	−2.55	313 (93), 298 (100), 283 (34), 270 (14), 255 (19),239 (6), 226 (83), 211 (43)	Luteolin-3′,4′-dimethyl ether	C_17_H_14_O_6_	[30]
27	9.19	309.0752	309.0763	−3.56	309 (100), 294 (25), 266 (29), 250 (10), 249 (30), 148 (13)	Hoslundal	C_18_H_14_O_5_	-
28	9.34	339.0855	339.0868	−3.83	339 (24), 324 (14), 310 (16), 309 (100), 281 (13), 253 (8), 209 (5)	Ambanol	C_19_H_16_O_6_	-
29	9.46	267.0651	267.0657	−2.25	252 (100), 224 (3), 223 (55), 208 (11), 195 (17), 135 (1), 132 (24), 91 (2)	Formononetin *	C_16_H_12_O_4_	[33]
30	9.77	353.1013	353.1025	−3.40	353 (100), 338 (40), 323 (71), 308 (32), 293 (17), 279 (73)	Ambonone	C_20_H_18_O_6_	-
31	9.98	323.0911	323.0919	−2.47	323 (100), 308 (62), 293 (61), 265 (32), 249 (26), 237 (16), 221 (4), 145 (14)	Neoraunone	C_19_H_16_O_5_	-
32	10.34	353.1014	353.1025	−3.11	353 (100), 338 (33), 323 (73), 308 (38), 293 (14), 279 (69)	Ambonone isomer	C_20_H_18_O_6_	-
Flavonoid Glycosides								
9	6.05	431.0963	431.0978	−3.48	431 (52), Y_0_^−^: 269.0446 (26), [Y_0_^−^H]^−•^: 268 (100), 241 (1), 224(4), 135 (1)	Genistin	C_21_H_20_O_10_	[37]
10	6.25	415.1017	415.1029	−2.89	415 (26), Y_0_^−^: 253.0494 (32), [Y_0_^−^H]^−•^: 252 (100), 223 (1)	Daidzin	C_21_H_20_O_9_	[38]
16	7.45	445.1124	445.1134	−2.25	Y_0_^−^: 283.0612 (100), 268 (8), 267 (1), 255 (16), 240 (2), 239 (2), 133 (40)	Glycitin	C_22_H_22_O_10_	[38]

* Identification of these compounds was confirmed by comparison with the standards (Supplementary Materials Figure S2).

## Data Availability

Data is contained within the article and supplementary material.

## Supplementary Materials

The following are available online: Figure S1: UV spectra of compound **17** (i), compound **18** (ii), compound **23** (iii) and compound **24** (iv); Figure S2: Comparison between the mass spectra of crude root extract (A) and crude root extract spiked with isoflavones standards (B); Figure S3: Proposed fragmentation pathway of compound **20** (3′,4′-dimethoxyflavonol); Figure S4: Proposed fragmentation pathway of compound **22** (Diosmetin); Figure S5: Proposed fragmentation pathway of compound **26** (luteolin-3′,4′-dimethyl ether); Figure S6: Proposed fragmentation pathway of compound **27** (Hoslundal); Figure S7: Proposed fragmentation pathway of compound **29** & **31** (Ambanone and its isomer); Figure S8: Proposed fragmentation pathway of compound **30** (Neoraunone); Figure S9: Proposed fragmentation pathway of compound **28** (Ambanol); Figure S10: (A) LC-DAD and (B) LC-MS base peak chromatogram profiles of *Clitorea ternatea* crude root extract in positive mode; Table S1: Metabolite profiles of *Clitorea ternatea* root extract in positive MS/MS; Table S2: Yield of aqueous, ethyl acetate, 50% methanol and hexane fractions from SPE.

## Sample Availability

Samples of the compounds are not available from the authors.
