# Supplementary material for: Clitorienolactones and Isoflavonoids of Clitorea ternatea Roots Alleviate Stress-Like Symptoms in a Reserpine-Induced Zebrafish Model
Source: Molecules. 2021 Jul 7;26(14):4137. doi: 10.3390/molecules26144137 (PMC8304078; doi:10.3390/molecules26144137)
Supplement: Supplementary file 1 [file molecules-26-04137-s001.zip › molecules-1234074-supplementary.pdf]

molecules

MDPI

Supplementary Materials

Clitorienolactones and Isoflavonoids of *Clitorea ternatea* Roots Alleviate Stress-Like Symptoms in Reserpine-Induced Zebrafish Model

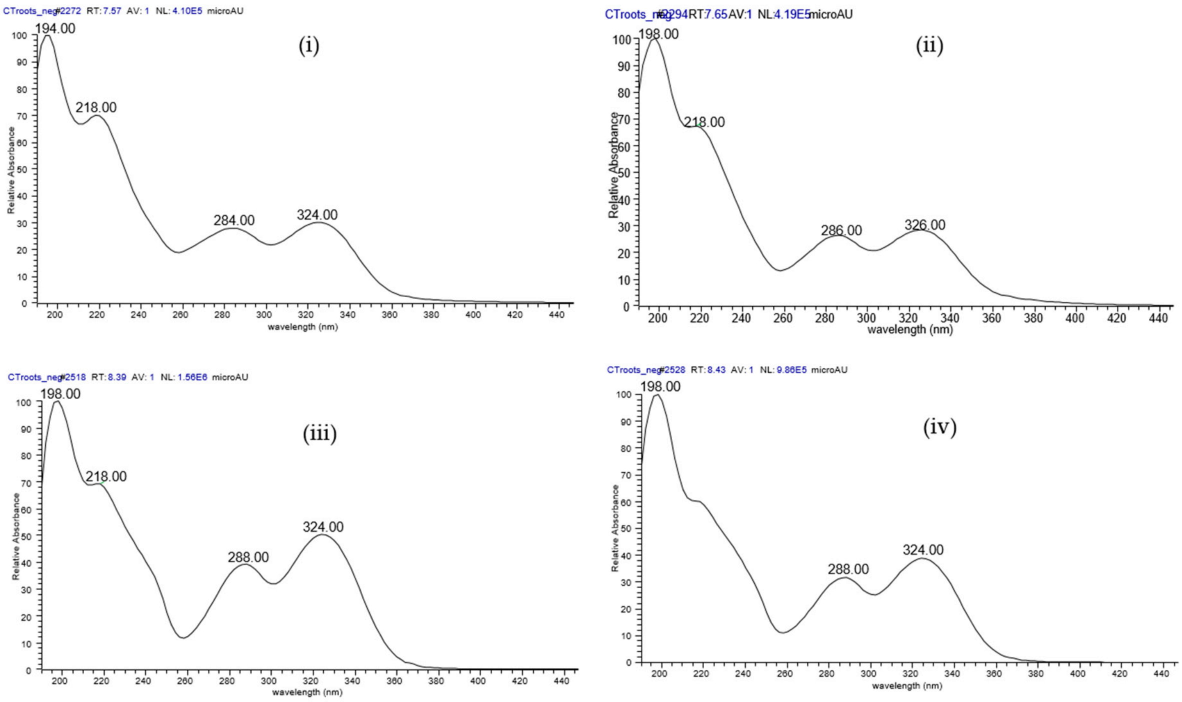

Figure S1. UV spectra of compound **17** (i), compound **18** (ii), compound **23** (iii) and compound **24** (iv)

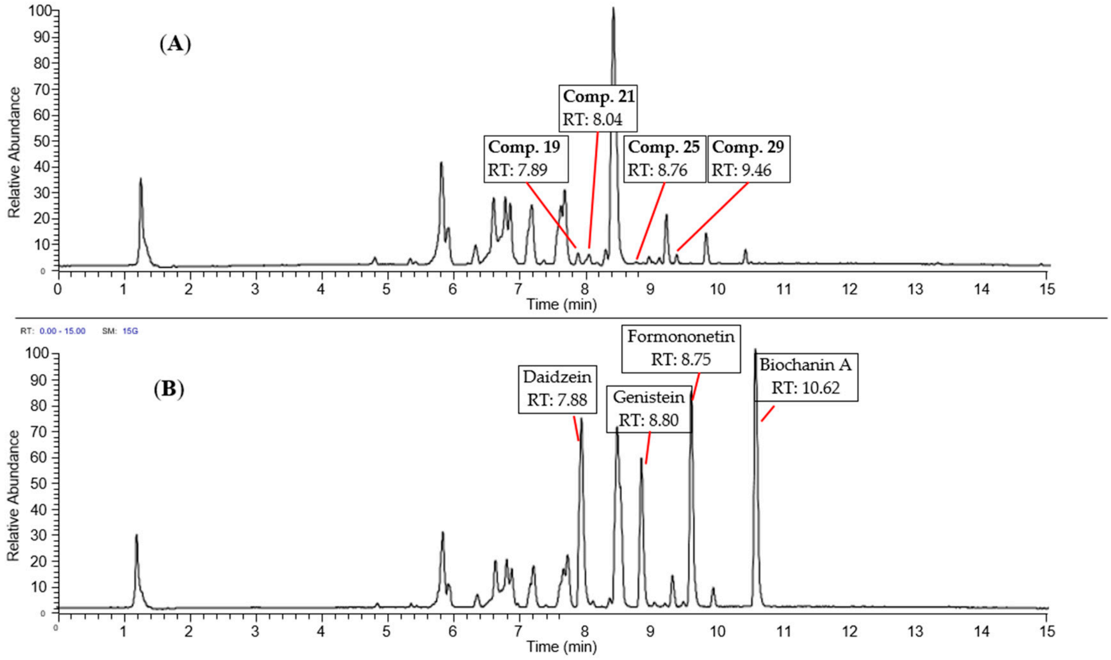

Figure S2. Comparison between the mass spectra of crude root extract (A) and crude root extract spiked with isoflavones standards (B).

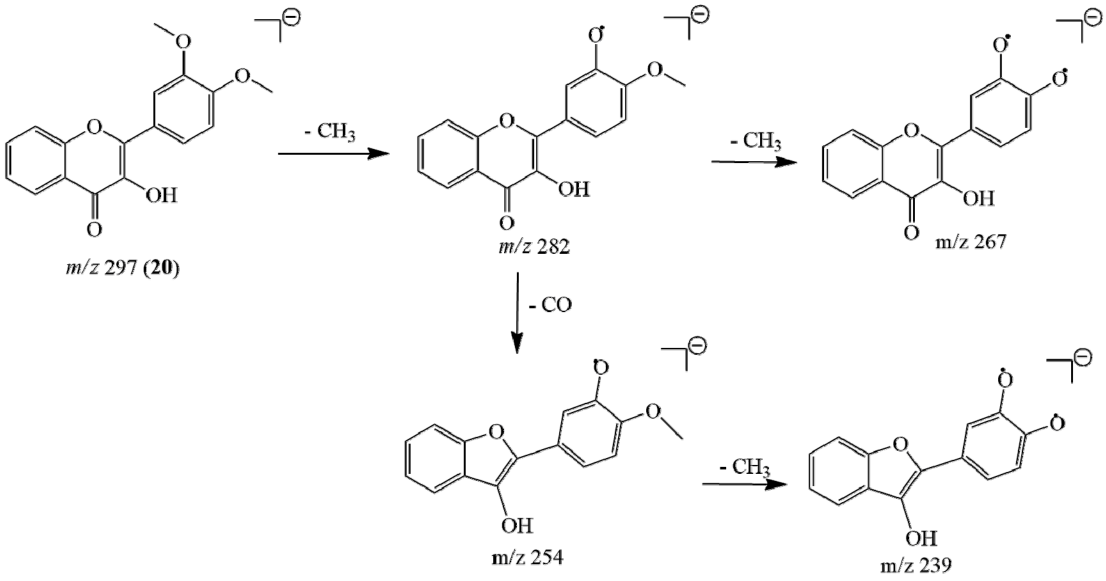

Figure S3. Proposed fragmentation pathway of compound **20** (3′,4′-dimethoxyflavonol).

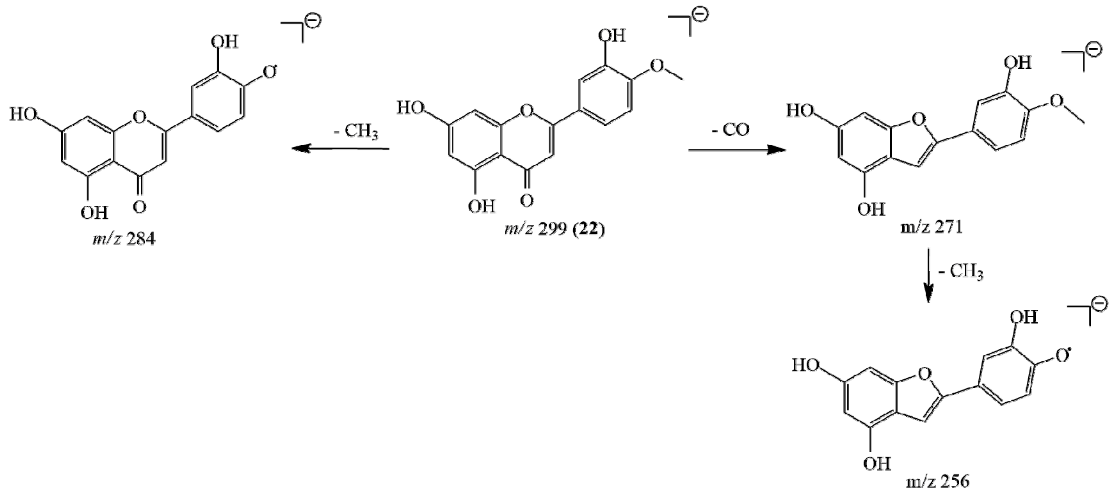

Figure S4. Proposed fragmentation pathway of compound **22** (Diosmetin).

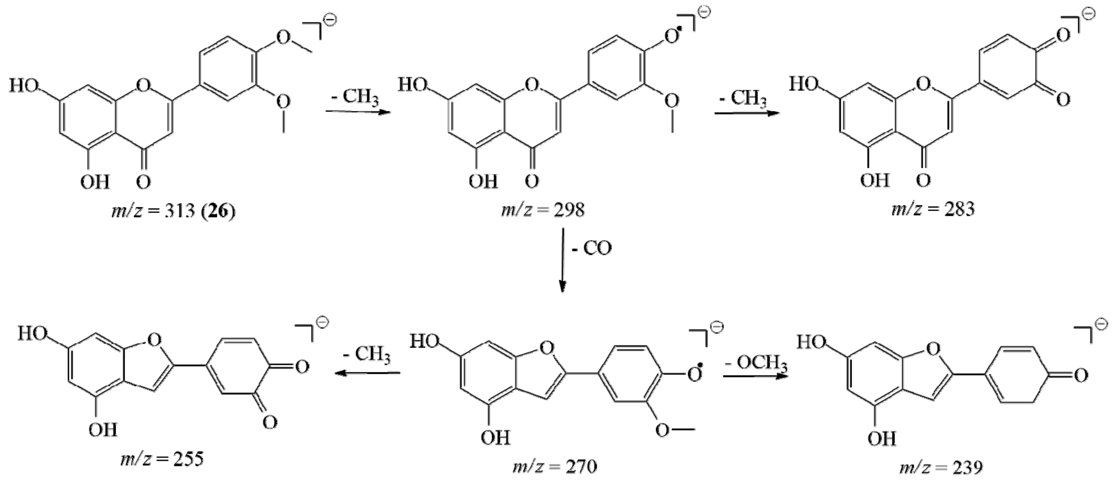

Figure S5. Proposed fragmentation pathway of compound **26** (luteolin-3′,4′-dimethyl ether).

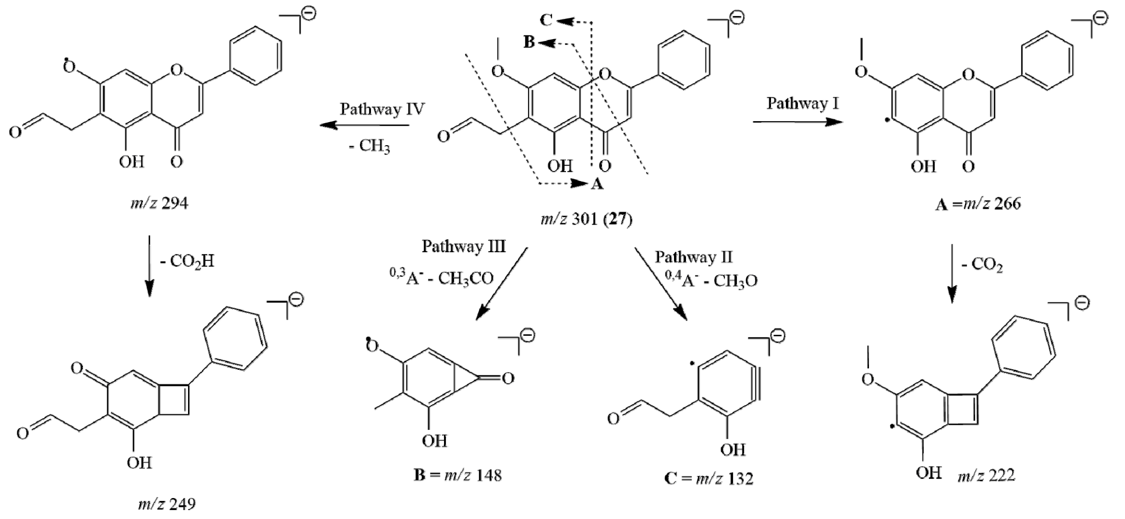

Figure S6. Proposed fragmentation pathway of compound **27** (Hoslundal).

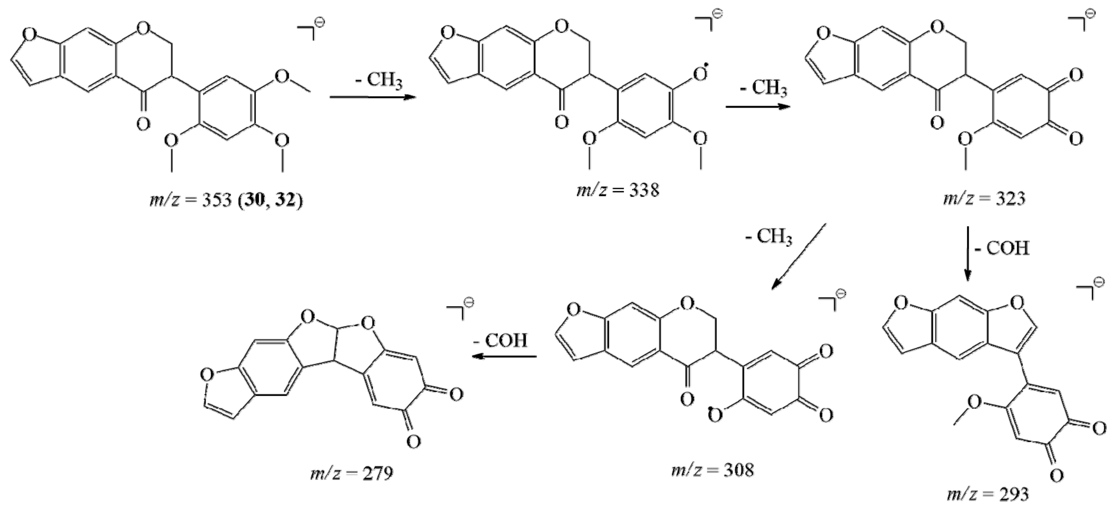

Figure S7. Proposed fragmentation pathway of compound **29** & **31** (Ambanone and its isomer).

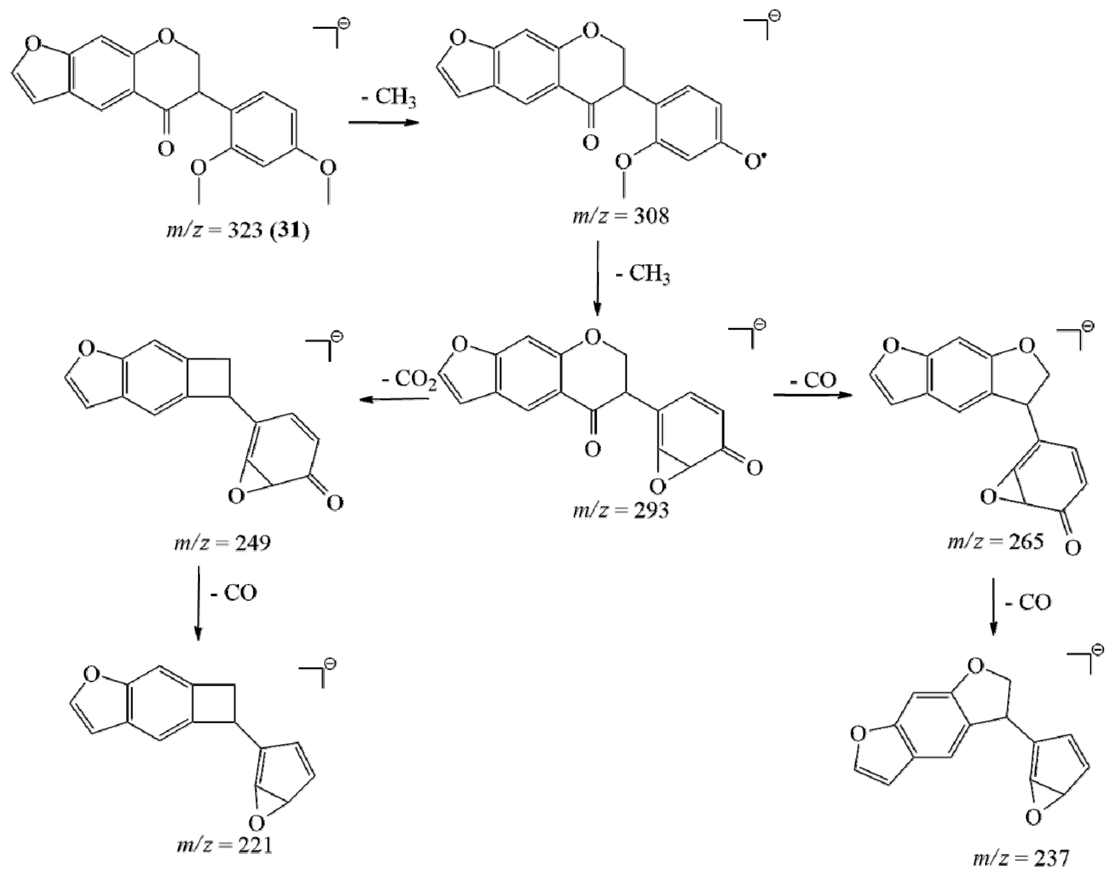

Figure S8. Proposed fragmentation pathway of compound **30** (Neoraunone).

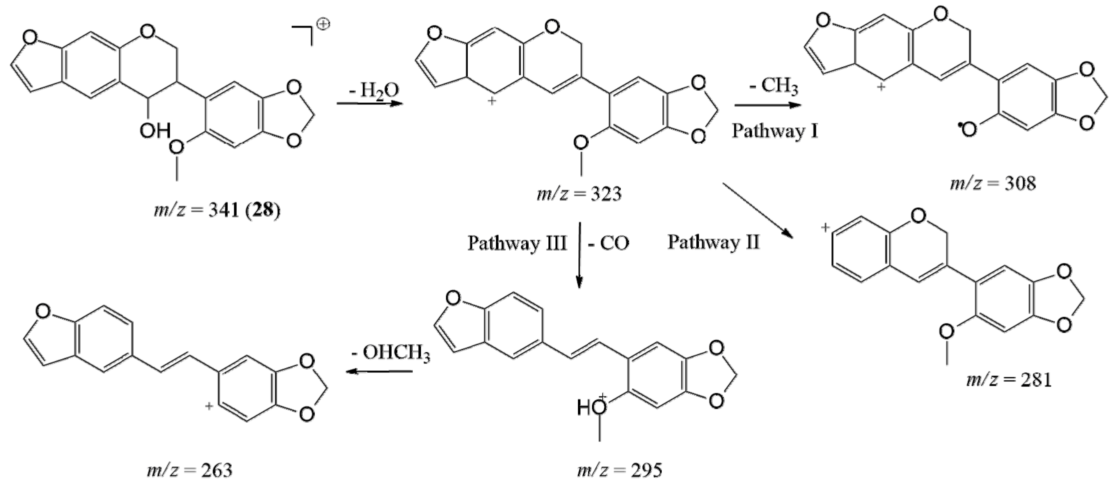

Figure S9. Proposed fragmentation pathway of compound **28 (**Ambanol).

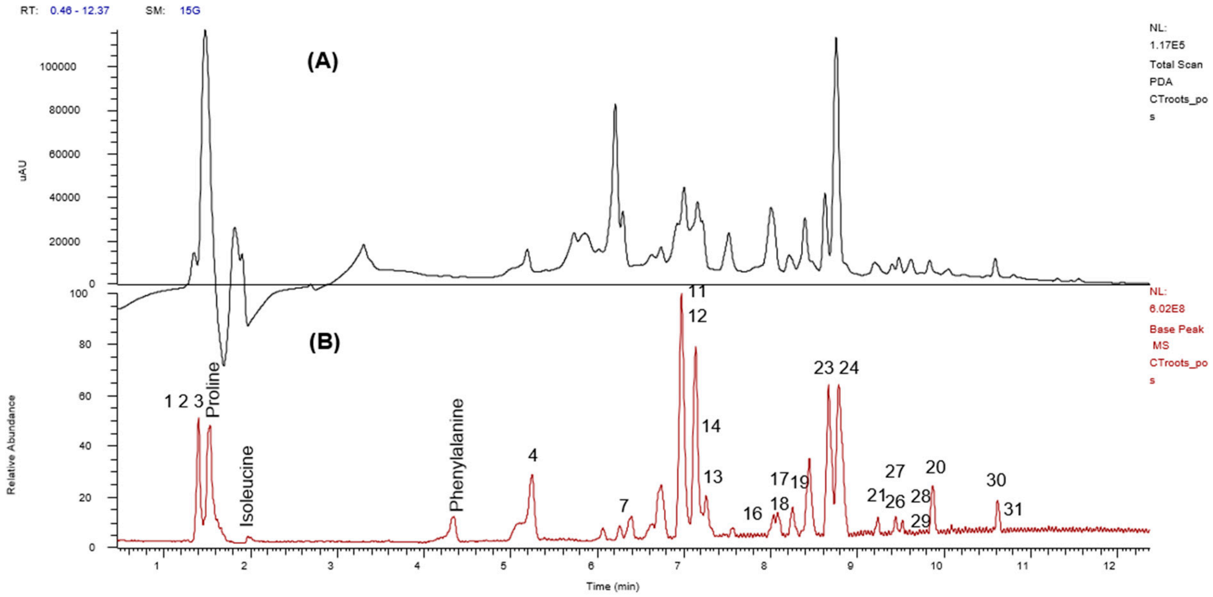

Figure S10. (A) LC-DAD and (B) LC-MS base peak chromatogram profiles of *Clitorea ternatea* crude root extract in positive mode.

Table S1. Metabolite profiles of *Clitorea ternatea* root extract in positive MS/MS.

No	Retention time (RT) (min)	Parent ion experimental (*m/z*)	Parent ion theoretical (*m/z*)	Error (ppm)	MS/MS fragment ions (intensity, %)	Tentative identification	Molecular formula	Source
					Amino acids & Carboxylic acids			
1	1.41	156.0751	156.0773	−14.09	156 (25), 138 (2), 110 (100), 95 (5), 83 (3)	Histidine	$C_6H_9N_3O_2$	[45]
2	1.4	175.1169	175.1195	−14.85	175 (100), 158 (19), 157 (5), 130 (22), 116 (57), 112 (6) 70 (84), 60 (61)	Arginine	$C_6H_{14}N_4O_2$	[45,46]
3	1.4	133.0593	133.0613	−15.03	133 (17), 116 (17), 88 (16), 87 (61), 75 (1), 74 (100), 70 (4)	Asparagine	$C_4H_8N_2O_3$	[45]
-	1.54	116.0695	116.0711	−13.78	116 (34), 70 (100)	Proline	$C_5H_9NO_2$	[45,46]
-	1.97	132.1005	132.1024	−14.38	132 (3), 113 (3), 87 (4), 86 (100), 72 (5), 69 (7)	Isoleucine	$C_6H_{13}NO_2$	[45,47]
-	4.36	166.0844	166.0868	−14.45	166 (4), 149 (1), 131 (3), 121 (6), 120 (100), 103 (3), 84 (12)	Phenylalanine	$C_9H_{11}NO_2$	[39,40,45]
4	5.23	205.0947	205.0977	−14.63	188 (100), 159 (8), 146 (58), 144 (10), 132 (4), 118 (7)	Tryptophan	$C_{11}H_{12}N_2O_2$	[45]
					Free Clitorienolactones			
17	8.07	299.0877	299.0919	−14.04	299 (18), 281 (12), 253 (22), 239 (13),193 (4), 107 (100)	Clitorienolactone D	$C_{17}H_{14}O_5$	[22]
18	8.08	329.0979	329.1025	−13.98	329 (3), 251 (2), 205 (2), 177 (2) 138 (6), 137 (100)	Clitorienolactone C	$C_{18}H_{16}O_6$	[22]
23	8.78	313.1033	313.1076	−13.73	313 (66), 295 (64), 267 (65), 253 (100), 236 (15), 207 (12), 147 (16), 107 (58)	Clitorienolactone B	$C_{18}H_{16}O_5$	[22]
24	8.83	343.1134	343.1181	−13.70	343 (21), 325 (11), 283 (10), 265 (7), 219 (17), 201 (7), 177 (14), 175 (7), 137 (100)	Clitorienolactone A	$C_{19}H_{18}O_6$	[22]
					Clitorienolactone glycosides			
7	6.26	637.2047	637.2132	−13.33	**Y_1^+** : 475 (100), **Y_0^+** : 313 (74), 295 (47), 267 (41), 253 (49), 207 (10), 107 (19)	Clitorienolactone B 4-O-di-hexoside	$C_{30}H_{36}O_{15}$	-

11	7.03	461.1385	461.1448	−13.66	461(2), **Y₀⁺** : 299 (50), 281 (11), 253 (13), 107 (100)	Clitorienolactone D 4-*O*-hexoside	$C_{23}H_{24}O_{10}$	-
12	7.08	491.1489	491.1553	−13.03	491 (0.3), **Y₀⁺** : 329 (17), 251 (1), 205 (2), 177 (1), 137 (100)	Clitorienolactone C 4-*O*-hexoside	$C_{24}H_{26}O_{11}$	-
13	7.25	505.1642	505.1710	−13.46	505 (18), **Y₀⁺** : 343 (35), 325 (16), 283 (10), 219 (18), 177 (14), 137 (100)	Clitorienolactone A 4-*O*-hexoside	$C_{25}H_{28}O_{11}$	-
14	7.20	475.1542	475.1604	−13.05	475 (48), **Y₀⁺** : 313 (100), 295 (55), 267 (51), 253 (73), 207 (14), 147 (11), 107 (32)	Clitorienolactone B 4-*O*-hexoside	$C_{24}H_{26}O_{10}$	-
					Flavonoid aglycones			
19	8.31	255.0621	255.0657	−14.11	255 (100), 227 (2), 213 (1), 199 (5), 137 (3)	Daidzein	$C_{15}H_{10}O_4$	[41]
20	9.89	299.0878	299.0920	−14.04	299 (100), 284 (12), 243 (2)	3′,4′-dimethoxyflavonol	$C_{17}H_{14}O_5$	-
21	9.25	285.0723	285.0763	−14.03	285 (100), 243 (2), 229 (5), 215 (16), 187 (4), 151 (6)	Glycitein	$C_{16}H_{12}O_5$	-
26	9.46	315.0826	315.0868	−13.33	315 (100), 300 (4), 255 (4), 175 (5), 167 (6)	Luteolin-3′,4′-dimethyl ether	$C_{17}H_{14}O_6$	-
27	9.54	311.0876	311.0919	−13.82	311 (100), 293 (9), 278 (3), 265 (7), 151 (4), 121 (36)	Hoslundal	$C_{18}H_{14}O_5$	-
28	9.66	341.0979	341.1025	−13.48	341 (100), 323 (5), 308 (8), 295 (4), 281 (9), 263 (9), 235 (5),121 (30)	Ambanol	$C_{19}H_{16}O_6$	-
29	9.79	269.0777	269.0814	−13.75	269 (100), 254 (3), 237 (1), 213 (3), 151 (1), 107 (1)	Formononetin	$C_{16}H_{12}O_4$	-
30	10.64	355.1134	355.1182	−13.52	355 (100), 337 (8), 324 (7), 322 (4), 295 (4), 278 (7), 121 (33)	Ambonone	$C_{20}H_{18}O_6$	-
31	10.82	325.1030	325.1076	−14.15	325 (100), 307 (13), 292 (6), 279 (8), 264 (5), 248 (4), 121 (38)	Neoraunone	$C_{19}H_{16}O_5$	-
					Flavonoid glycosides			
16	7.88	447.1234	447.1291	−12.75	447 (14), **Y₀⁺** : 285 (100), 215 (7), 151 (3)	Glycitin	$C_{22}H_{22}O_{10}$	-

Table S2. Yield of aqueous, ethyl acetate, 50% methanol and hexane fractions from SPE.

**Fractions**	**Yield (mg)**
Aqueous	65
Ethyl acetate	143
50% methanol	398
Hexane	47
